# Tourism and Livable Towns Beyond the Coronavirus Disease 2019: A Case Study for Chongqing, China

**DOI:** 10.3389/fpubh.2020.624519

**Published:** 2020-12-18

**Authors:** Ke Su, Chao Zhou

**Affiliations:** ^1^Yangtze Economic Research Center, Chongqing Technology and Business University, Chongqing, China; ^2^Institute of Finance and Economics, Chongqing College of Electronic Engineering, Chongqing, China; ^3^Chongqing Institute for International Strategies, Sichuan International Studies University, Chongqing, China

**Keywords:** COVID-19 outbreak, tourist towns, characteristic Chinese landscape, ecologically livable cities, Chongqing

## Abstract

Based on the data of 812 small towns in Chongqing, China, this paper attempts to conduct an empirical analysis on whether tourist towns with excellent natural environment, policy advantage, and market preference are more ecologically livable than ordinary small towns. It is found that as a whole, tourist towns are indeed more ecologically livable than ordinary small towns. Also, from the perspective of grading, both the national and provincial tourist towns have the advantage of ecological livability, but the advantage of national ones is more prominent. Furthermore, the ecological livability of tourist towns is affected by location advantage and policy inclination. The implications of the results are discussed following the outcomes of the coronavirus disease 2019 outbreak. The suggestions beyond the coronavirus disease 2019 are also provided.

## Introduction

The Ministry of Housing and Urban–Rural Development and the National Tourism Administration of China have jointly evaluated and elected national tourist towns since 2010. To protect tourist towns with characteristic Chinese landscape (i.e., tourist towns), further promote the rural human environment and tourism of those towns, 2010, 2011, and 2015, the first, second, and third batches of national tourist towns were selected, totaling 372 towns (1). In addition to the evaluation at the national level, provincial governments also evaluated provincial tourist towns^1^. Taking Chongqing as an example, in 2013, the Chongqing Municipal Government evaluated and selected the first batch at the provincial level, totaling 10 towns (2).

The outbreak of the coronavirus disease 2019 (COVID-19) showed that many cities are yet to meet the livability criteria and that there are urgent needs for public health systems with resilience at both national and local levels. The COVID-19 pandemic has imposed some critical challenges for cities in containing the epidemic. Achieving sustainable development goals requires cities to transform by creating spaces that foster urban mobility, resilience, and equality. Tourism may help to achieve these goals. Note that COVID-19 affects the Chinese economy. Domestic tourism has also been affected by COVID-19, given that there are tremendous precautions for visitors from China. Less mobility may negatively affect tourism demand, and domestic tourism may be a substitute for international tourism during COVID-19 (3), and this may help the natural environment of touristic towns, such as Chongqing.

The population is the core of urban development, and ecological livability is one of the essential conditions to retain and attract population, no matter for tourist towns or ordinary small towns. A large number of studies have pointed out that there are significant differences between urban and rural areas in the main aspects of ecological livability—necessary public service facilities and natural environment (4–8). Under the long-term urban–rural dual system, inequality in these aspects is an important factor causing the widening gap between urban and rural areas (9, 10). Although there is a lot of literature on ecological livability, the research field is mainly concentrated in cities, and little attention has been paid to small towns (11–15). Only a few scholars, like Yang and Wang (16), have carried out relevant research. Because of the fascinating natural landscape and rich historical and cultural deposits, tourist towns have become the treasure of more than 30,000 small towns in China. In the process of Rural Revitalization and New Urbanization in China, tourist towns will play a crucial role in regional industrial integration and green development due to their advantage in promoting the tourism industry, government support, and market preference. One of the critical issues affecting the long-term development of tourist towns is whether these towns with the advantage of ecological livability are suitable for the development of the tourism industry and the residing population. This issue is not only related to the effectiveness of relevant government policies but also has a great impact on the strategy of Rural Revitalization and the construction of New Urbanization. Therefore, it has profound practical significance and theoretical connotation to investigate whether this particular group of small towns has the advantage of ecological livability.

The rest of the paper is organized as follows. Section Research Hypotheses provides the research hypotheses with different characteristics of tourism towns. Section Measuring Ecological Livability, Data, and Empirical Model explains the measurement of ecological livability, data, and empirical model. Section Empirical results discusses the empirical results, and Section Conclusion concludes.

## Research Hypotheses

### Government-Led Urbanization and the Development of Small Towns

In China's urbanization system, governments dominate the allocation of resources. Specifically, the government has direct jurisdiction over the establishment of cities and towns, the approval of land use, the change of land function, the construction of infrastructure, and many other aspects. Li et al. (17) sum up six modes of promoting urbanization in China: establishing development zones, building new districts and new towns, urban expansion, old city transformation, building central business districts, township, and village industrialization. In this sense, the government is undoubtedly the most influential designer and executor. The competition mechanism among local governments has played an essential role in the continuous and high-speed growth of China's economy (18). Economic growth is regarded as one of the most critical “competition yardsticks” among local governments (19). To achieve faster economic growth, local governments often take measures, including attracting investment and cultivating profitable industries. Unlike the eastern region in China, the western region is relatively backward in location advantage and scarce in resources endowment. Simultaneously, due to policy factors such as environmental protection, the economic development of the western region faces some policy constraints. Therefore, the tourism industry has become an essential pillar for local governments in the western region to develop its economy. Because of its geographical location, the natural environment, and ethnic customs in the western region are well-preserved. Under the government-led urbanization system, more resources will be invested in the tourism industry. Regions with high-quality tourism resources will receive preferential support, including funds, land, and other policies from the governments at a higher level and then acquire an advantage in the development of the tourism industry (20). Similar to interprovincial competition, local governments within provinces and municipalities will also tilt more resources toward industries and areas with development advantages.

Although governments play a leading role in the process of urbanization, the impact of the market on resource allocation is also enormous. In the market allocation mechanism, population, and other production factors are all seeking to maximize benefits. From the view of tourism development, with the growth of the economy and urbanization, an increasing number of urban residents are more eager to get a higher level of tourism perception (21), which is in contradiction with the types and quality of products provided in the current tourism market. At present, there are many problems in China's tourist attractions, ranging from overcommercialization to serious homogeneity. The problems of historical tourist towns are particularly prominent (20). Tourist towns are natural carriers for the development of rural tourism (22). Therefore, as the treasure of small towns in China, tourist towns will attract many production factors to upgrade the infrastructure and natural landscape. Besides, in China's current urbanization system, the government's support and guidance often mean that tourist towns will enjoy the support of land and fiscal policy. Based on the earlier analysis from the perspective of government and market, this paper puts forward the following hypotheses:

H1: Because of a better natural environment, policy support, and the favor of the market, tourist towns have a higher level of ecological livability than ordinary small towns.

H2: In regions with higher tourism dependence, the advantage in the ecological livability of tourist towns is more significant.

### Government Rating: Self-Selection and Resource Guidance of Small Towns

China's tourism resources are usually graded. As for the evaluation level, it is generally divided into national and provincial ones. At the national level, due to the enormous number of selected objects and high requirements, it is more difficult for tourism resources to be rated as national ones (23). To develop the local economy, local governments often elect resources with a more competitive advantage to participate in the national evaluation and then enhance the popularity of tourism resources in their jurisdiction.

Similar to the national evaluation, in provincial evaluation, the lower level government will choose its high-quality tourism resources for evaluation out of similar motives to enhance the economic competitiveness of its region. In terms of the quality of tourism resources, the rating mechanism has formed self-selection of tourism resources within regions. From the public perspective, there are some differences in the degree of public trust between the central government and local ones (24). The public has a higher degree of trust with the central government, whereas the degree of trust with local governments is lower. In this sense, compared with tourist towns at the provincial level, the national ones can gain more public trust. In this context, whether for the needs of local governments to develop their economy or for enterprises to cater to the market and earn profits, more resources will be tilted to the national tourist towns. Based on the analysis of the selection mechanism of tourist towns and the investment motivation of local governments and enterprises, this paper puts forward the following research hypothesis:

H3: Compared with the provincial tourist towns, the advantage of national tourist towns in ecological livability is more prominent.

### Three Gorges Project: State Support and Development of Small Towns in the Reservoir Area

The Three Gorges Project is the largest project ever built in the world. Two hundred seventy-seven towns have been inundated owing to the construction of the Three Gorges Project, and 1.13 million migrants have been relocated (25). For the inundated towns, governments mainly adopt the way of relocation and reconstruction. Due to the large amount of funds invested in infrastructure construction, coupled with land, tax, and other policy support, the overall development level of relocated and reconstructed towns has been dramatically improved (26). Not only in the construction process of the Three Gorges Project but also after the completion of the project, the Three Gorges Reservoir Area has enjoyed tremendous support in finance and policy. Taking the “Three Gorges Follow-up Work Plan” approved by the State Council of China in 2011 as an example, the central government planned to invest 123.8 billion yuan (~19 billion US $) in the Three Gorges Reservoir Area from 2011 to 2020. Among them, the investment in the Chongqing Reservoir Area of the Three Gorges is approximately 80 billion yuan (~12.6 billion US $) (27). In addition to financial support, the government has also given great support to the Three Gorges Reservoir Area in aspects including land, industry, and natural environment. Despite tremendous support gained at the national level, there are still some problems in the Three Gorges Reservoir Area, such as the hollowing of industrial structure and low level of economic development caused by the relocation of enterprises and remote location (28). Because of its unique geographical location, the natural environment of the Three Gorges Reservoir Area is fragile, which has attracted lots of attention from the government and society (29). Because the tourism industry is friendly to the environment and the Three Gorges is also a world-famous scenic spot, the tourism industry turns out to be an ideal industry for the Three Gorges Reservoir Area. Based on the earlier analysis, the following hypothesis has been put forward.

H4: Tourist towns located in the Chongqing reservoir area of the Three Gorges have a more significant advantage in ecological livability. The scarcity of industrial development resources makes the tourist towns abler to obtain a wide range of support from the government and the market.

## Measuring Ecological Livability, Data, and Empirical Model

### Measuring Ecological Livability

**Figure 1:**
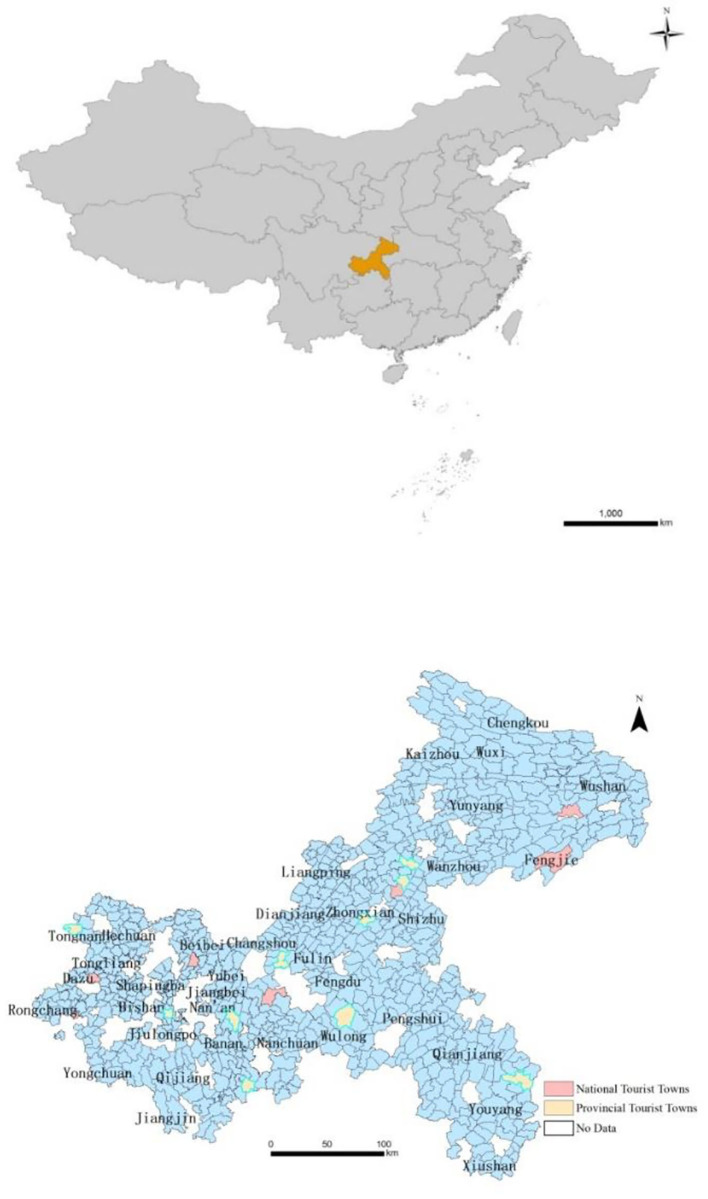
Study area.

Located in the upper reaches of the Yangtze River, Chongqing is a national historical and cultural city with a history of more than 3,000 years and precious tourism resources. Because of its unique geographical form and distinct development conditions, Chongqing has formed a comprehensive structure of “large city, large countryside, large mountain area, and large reservoir area.” It was designated as a pilot area of comprehensive urban–rural reform by the State Council of China in 2007. At the end of 2015, Chongqing had 38 districts and counties and 812 small towns. Among the two batches of national tourist towns released in 2010 and 2011, seven small towns were selected in Chongqing. In 2013, the Chongqing Municipal Government announced the first batch of provincial tourist towns, including 10 small towns (see Figure 1). It should be noted that none of the 10 small towns was previously rated as a national tourist town (27). To sum up, the research scope of this paper is 812 small towns in 38 districts and counties of Chongqing, including 17 tourist towns. The detailed list is shown in Table 1.

**Table 1:** List of touristic towns in Chongqing.

**National tourist towns**		
The first batch (2010)	Jing Guan, Wan Ling, Xing Long	3
The second batch (2011)	Bao Ding, Bai Di, Shi Bao, Lin Shi	4
**Provincial tourist towns**		
The first batch (2013)	Dong Wenquan, You Shuihe, Xian Nvshan, Wu Lingshan, Hei Shan, Bai Shiyi, Cong Kan, Chang Shouhu, Xin Sheng, Gan Ning	10
Total		17

Referring to the research results of Mahmoudi et al. (12), Yang and Wang (16), and based on the principles of data availability, this paper constructs an evaluation index system of ecological livability of 812 small towns in Chongqing from two dimensions, namely the natural environment and human and social environment. In this paper, the ecological livability of small towns in the natural environment is estimated by six indicators, such as sewage treatment rate and waste treatment rate, which is more diverse than previous literature. Because the entropy method has the advantage of objectivity and being able to avoid overlapping information among various indicators, this paper uses the entropy method to measure the ecological livability of 812 small towns in Chongqing based on data normalization. Detailed information like indicators, entropy, redundancy, and weight are shown in Table 2.

**Table 2:** Evaluation index system of ecological livability of 812 small towns, Chongqing.

**Primary indices**	**Secondary indices**	**Information entropy**	**Redundancy**	**Weight**
Livability of natural environment	Sewage treatment rate (%)	0.9342	0.0658	0.0634
	Waste disposal rate (%)	0.8192	0.1808	0.1701
	Harmless treatment rate (%)	0.9762	0.0238	0.0229
	Green coverage in built-up areas (%)	0.9214	0.0786	0.0739
	Green space rate in built-up area (%)	0.9161	0.0839	0.0808
	Per capita park green space area (km^2^)	0.9365	0.0635	0.0612
Livability of humanistic and social environment	Per capita road area in the built-up area (km^2^)	0.9348	0.0652	0.0628
	Number of public toilets for 10,000 people	0.9525	0.0475	0.0458
	Water penetration (%)	0.9192	0.0808	0.0779
	Gas penetration (%)	0.9407	0.0593	0.0558
	Number of hospitals for 10,000 people	0.9605	0.0395	0.0381
	Number of beds for 10,000 people	0.9455	0.0545	0.0525
	Number of General primary schools for 10,000 people	0.9906	0.0094	0.0090
	Number of ordinary middle schools for 10,000 people	0.9497	0.0503	0.0484
	Urban per capita residential area (square meters)	0.9416	0.0584	0.0563
	Per capita residential area in rural areas (square meters)	0.9158	0.0842	0.0811

It is important to note that we used three sources of data. The first is the *Basic Data of Towns and Townships in Chongqing 2016*, released by the Chongqing Urban and Rural Construction Committee. The *Basic Data of Towns and Townships in Chongqing 2016* contains 23 indicators such as gross domestic product (GDP), built-up area, and population of 812 small towns in Chongqing, which is comprehensive and authoritative statistical data on small towns in Chongqing. The second one is the list of tourist towns, which include the first and second batches of national tourist towns jointly released by the Ministry of Housing and Urban–Rural Development and the National Tourism Administration of China in 2010 and 2011, respectively. The first batch of tourist towns was released by the Chongqing Municipal Government in 2013. Although the third batch was released in July 2015, this paper does not include it in the research scope due to the short period of validity. The third one is the statistical yearbooks, and the last one is Baidu Map. Statistical yearbook data come from the Chongqing Statistics Bureau, including *Chongqing Statistical Yearbook 2016* and a total number of 38 statistical yearbooks of districts and counties in Chongqing.

### Data and Empirical Model

The ecological livability of small towns calculated earlier is between 0 and 1, which has non-negative truncation characteristics. For estimating such constrained explanatory variables, the general mixed effect model will distort the results. Therefore, we adopt the Tobit model to conduct the regression analysis. To reduce the influence of regression errors caused by missing variables, we take various indicators, including economic development, population urbanization, land urbanization, and population size as control variables based on the relevant research of urban economics (30, 31). It is important to note the previous literature regarding the influencing factors of human settlements in small towns. Besides, due to differences in resource endowment, government support, and other aspects, there may be some gaps in ecological livability between national and provincial tourist towns. Therefore, this paper will further explore the possible differences between the two. In this sense, this paper constructs the following econometric models:

(1) $$\begin{array}{l} Live=\alpha_{0}+\alpha_{1}towns+\alpha_{2}\ln\;pgdp+\alpha_{3}\ln\;distance\\ \;+\alpha_{4}\ln\;popur+\alpha_{5}\ln\;landur+\alpha_{6}\ln\;pop+\alpha_{7}central+\varepsilon \end{array}$$

(2) $$\begin{array}{l} Live=\alpha_{0}+\alpha_{1}towns_{1}+\alpha_{2}towns_{2}+\alpha_{3}\ln\;pgdp\\ \;+\alpha_{4}\ln\;distance+\alpha_{5}\ln\;popur+\alpha_{6}\ln\;landur\\ \;+\alpha_{7}\ln\;pop+\alpha_{8}central+\varepsilon \end{array}$$

In Equation (1), the explained variable () indicates the ecological livability of small towns, which is calculated from Table 2. The core explanatory variable (*towns*) is the dummy variable of the tourist towns. α_1_ is the coefficient mainly concerned in the paper. In Equation (2), the core explanatory variable (*towns*) is further divided into national tourist towns and provincial ones. There are six controlled indicators in the models discussed.

Per capita GDP (*pgdp*) is mainly used to measure the economic development of small towns. Economic development and ecological livability are often closely linked. To promote economic development, local governments perhaps will increase their tolerance of environmental pollution; on the other hand, a higher level of economic development provides support for the construction and maintenance of environmental infrastructure in small towns (32). It should be noted that although there may be a certain degree of multicollinearity between per capita GDP and the central town, it will not affect the estimation results of the core explanatory variable (33).

Distance from district and county governments (*distance*) refers to the closest distance between small towns and district and county governments. It is mainly used to analyze the radiation effect of core urban areas on the ecological livability of small towns. In theory, small towns closer to the core urban areas can enjoy more spillover effects, so the economy and basic public service facilities are better. The data are obtained by using Baidu Map in June 2017.

Urban population (*popur*) is the population living in the built-up area of small towns. As an essential aspect of urbanization, population urbanization is a powerful driving force to promote the development of urban natural environments and basic public service facilities.

Land urbanization (*landur*) is the urban built-up area of small towns. The expansion of the built-up area will hurt the natural environment and will also increase the financial burden of upgrading necessary public service facilities.

Population size (*pop*) refers to the permanent population of small towns. Similar to the impact of the built-up area, the increase of population size will theoretically hinder the improvement of the ecological livability of small towns.

Central town (*central*) is a dummy variable that indicates whether a small town is a central town or not. The central town refers to the town with better location advantage, a stronger economy, better infrastructure, and greater development potential. Due to a higher level of economic development and better basic public service facilities, the central town's ecological livability may be higher. We aim to exclude the possibility that small towns are more ecologically livable because they are central towns rather than tourist towns. Therefore, we add this dummy variable.

It should be pointed out that the data of per capita GDP, population urbanization, land urbanization, population size, and central town come from the *Basic Data of Towns and Townships in Chongqing 2016*. Besides, the total tourism revenue and GDP of each district and county in the following analysis are derived from the *Statistical Yearbook of 2016* of each district and county. All control variables, except dummy variables, are logarithmically processed. is the random error term. The descriptive statistics of related variables are shown in Table 3.

**Table 3:** Descriptive statistics.

**Variable**	**Symbol**	**Unit**	**Observation**	**Mean**	**Std. dev**.	**Min**.	**Max**.
Ecological livability	*Live*	/	812	0.15	0.07	0.02	0.49
Tourist towns	*towns*	/	812	0.02	0.15	0	1
Per capita GDP	*pgdp*	10,000 yuan/person	812	2.14	4.50	0.08	82.00
Distance from district and county governments	*distance*	kilometer	812	41.24	24.30	0.50	146.30
Population urbanization	*popur*	10,000 people	812	0.80	1.19	0.01	11.46
Land urbanization	*landur*	Square kilometer	812	1.40	3.06	0.01	61.63
Population size	*pop*	10,000 people	812	2.80	2.19	0.11	22
Central town	*central*	/	812	0.13	0.34	0	1

## Empirical Results

### Results of Benchmark and Hierarchical Regression

Firstly, this paper analyzes whether tourist towns have better ecologically livable environment compared with ordinary small towns and then divides them into national and provincial tourist towns. As mentioned earlier, national tourist towns usually have a better natural environment than provincial ones. Besides, the differences between national and provincial titles probably have a significant impact on the attraction to tourists, which may drive local governments and tourism enterprises to invest more resources in national tourist towns. Therefore, national tourist towns can get more policy support and capital investment. In contrast, the resources for provincial tourist towns will be relatively limited, which results in the difference in ecological livability between national and provincial tourist towns. Out of this consideration, this paper will divide tourist towns into national and provincial tourist towns, and then, the test of Hypothesis 3 will be conducted from the perspective of grading. According to Table 1, there are a total of seven small towns in Chongqing rated as national tourist towns and 10 small towns rated as provincial ones. The regression results are shown in Table 4.

**Table 4:** Results of benchmark and hierarchical regression.

**Dependent variable: ecological livability of small towns**				
**Explanatory variables**	**(1)**	**(2)**	**(3)**	**(4)**
Tourist towns	0.053*** (0.016)	0.039** (0.015)		
National tourist towns			0.052** (0.024)	0.040* (0.024)
Provincial tourist towns			0.023** (0.011)	0.020** (0.009)
Per capita GDP		0.068*** (0.005)		0.072*** (0.005)
Distance from district and county governments (logarithm)		−0.028*** (0.004)		−0.028*** (0.003)
Population urbanization (logarithm)		0.006* (0.003)		0.007* (0.004)
Land urbanization (logarithm)		−0.002 (0.003)		−0.002 (0.003)
Population size (logarithm)		−0.012* (0.005)		−0.010** (0.005)
Central town		0.011*** (0.003)		0.011*** (0.003)
Constant	0.150*** (0.002)	0.252*** (0.014)	0.150*** (0.002)	0.252*** (0.014)
Observation number	812	812	812	812
Log likelihood	1,039.902	1,096.18	1,039.903	1,096.205
LR test	10.87	123.43	10.87	123.48

Values in parentheses are standard deviation, and ***, **, and * indicate the significant levels of 1, 5, and 10%, respectively.

Based on columns (1) and (2), we can notice that the coefficient of tourist towns is positive and significant at the level of 1%. This result shows that compared with ordinary small towns, tourist towns have a higher level of ecological livability due to government support, enterprise investment, or their pleasant natural environment. The results of columns (3) and (4) indicate that both national and provincial tourist towns have a higher level of ecological livability. Also, as previously analyzed, owing to the differences in natural environment and policy inclination, the advantage in the ecological livability of national tourist towns is more prominent than provincial ones. That is to say, Hypothesis 3 of this paper is valid.

In terms of the relationship between ecological livability of small towns and the distance from small towns to the district and county governments, the farther the distance is, the weaker the radiation effect produced on the ecological livability of small towns by the core urban areas will be, which is in line with the reality. Besides, the research conclusion of Combes et al. (34) on population urbanization promoting urban development has been confirmed at the level of small towns, namely population urbanization has indeed played a particular role in promoting the ecological livability of small towns. Unlike population urbanization, land urbanization hurts the ecological livability of small towns, but the impact is small and has not passed the significant test. The expansion of the population-scale does not improve the ecological livability of small towns. Instead, it plays a reverse role. This evidence is closely related to the fact that Chongqing has more mountains and fewer plains, and a large number of rural populations is sparsely scattered, thus restricting the improvement of necessary public service facilities. As for the relationship between the ecological livability and central towns, the results prove that the ecological livability of central towns is significantly better.

### Results Based on Tourism Dependence

In addition to economic development, the importance of tourism in the local economy is also a key factor affecting how much resources the local government will invest in protecting and developing tourist towns. The more significant the proportion of tourism revenue in the local economy, the stronger local governments' motivation to protect and develop tourist towns. In this sense, the higher the tourism dependence is, the more prominent the advantage of tourist towns in ecological livability will be; on the other hand, the advantage of regions with lower tourism dependence will not be significant. Based on this consideration, this paper takes the proportion of tourism revenue to GDP as a measure of tourism dependence so that the difference in ecological livability of tourist towns scattered in regions with various tourism dependence will be studied, which is also a test for the Hypothesis 2 proposed by this paper. Taking the median of tourism dependence of 38 districts and counties in Chongqing as the dividing line, this paper divides them equally into two groups of regions with low and high tourism dependence. The detailed results are shown in Table 5. Among the 19 districts and counties with low tourism dependence, there are 10 tourist towns, of which the number of national tourist towns is 4, and the number of provincial tourist towns is 6. Among the 19 districts and counties with high tourism dependence, there are seven tourist towns, where the number of national and provincial tourist towns is 3 and 4, respectively.

**Table 5:** Tourism dependence of 38 districts and counties in Chongqing.

**Category**	**District and County**	**National tourist towns**	**Provincial tourist towns**
Low tourism dependence	Yu Bei, Ba Nan, Fu Ling, Chang Shou, Feng Jie, Yun Yang, Wu Shan, Wu Xi, Wan Zhou, Kai Zhou, Zhong Xian, Feng Du, Wu Long, Shi Zhu	Xing Long, Shi Bao, Ling Shi, Bai Di	Dong Wenquan, Xian Nvshan, Chang Shouhu, Xin Sheng, Gan Ning
High tourism dependence	Jiang Bei, Sha Pingba, Jiu Longpo, Nan An, Bei, Da Dukou, Qi Jiang, Wan Sheng, Da Zu, Qian Jiang, He Chuan, Yong Chuan, Nan Chuan, Rong Chang, Tong Nan, Liang Ping, Cheng Kou, Dian Jiang, Xiu Shan, You Yang, Peng Shui	Jing Guan, Wan Ling, Bao Ding	You Shuihe, Hei Shan, Bai Shiyi, Cong Can

Based on the division of 38 districts and counties in Chongqing, this paper further investigates whether tourist towns are still more ecologically livable in regions with different tourism dependence. The regression results are shown in Table 6.

**Table 6:** Results based on the division of tourism dependence.

**Dependent variable: ecological livability of small towns**				
**Explanatory variables**	**(1)**	**(2)**	**(3)**	**(4)**
	**Regions with low tourism dependence**		**Regions with high tourism dependence**	
Tourist towns	0.025 (0.018)		0.048** (0.021)	
National tourist towns		0.036 (0.026)		0.057*** (0.020)
Provincial tourist towns		0.015 (0.025)		0.020** (0.008)
Per capita GDP	0.068*** (0.006)	0.069*** (0.005)	0.078*** (0.002)	0.080*** (0.004)
Distance from district and county governments (logarithm)	−0.037*** (0.005)	−0.037*** (0.005)	−0.015** (0.006)	−0.016*** (0.006)
Population urbanization (logarithm)	0.001 (0.005)	0.001 (0.005)	0.008 (0.007)	0.008 (0.007)
Land urbanization (logarithm)	−0.002 (0.005)	−0.002 (0.005)	−0.004 (0.005)	−0.004 (0.005)
Population size (logarithm)	−0.021*** (0.006)	−0.021*** (0.006)	−0.015** (0.006)	−0.015** (0.006)
Central town	0.015*** (0.004)	0.015*** (0.004)	0.009* (0.005)	0.009* (0.005)
Constant	0.292*** (0.018)	0.292*** (0.018)	0.221*** (0.024)	0.222*** (0.024)
Observation number	356	356	234	234
Log likelihood	521.103	521.275	343.347	343.785
LR test	74.93	75.28	41.00	41.88

Values in parentheses are standard deviation, and ***, **, and * indicate the significant levels of 1, 5, and 10%, respectively.

From the results of columns (1) and (2) in Table 6, it is noticeable that the advantage in the ecological livability of 10 tourist towns located in regions with low tourism dependence is not significant, which is consistent with the expected results. Furthermore, both the national and provincial tourist towns are no more ecologically livable than ordinary small towns. According to the results of columns (3) and (4), in regions with high tourism dependence, tourist towns have a remarkably higher level of ecological livability. From the perspective of grading, the advantage in the ecological livability of national tourist towns and provincial ones is significant. This result is consistent with Hypothesis 2. Also, this result proves that under the current urbanization development pattern in China, the development and construction of small towns are closely related to local governments' support.

### Results Based on the Division of Reservoir Area and Non-reservoir Area of the Three Gorges Project

As discussed earlier, compared with small towns in the non-reservoir area, the primary public service facilities of small towns in the reservoir area have been rebuilt or improved during the construction of the Three Gorges Project. Besides, those towns have received plenty of support in finance and policy from the central government and local governments after completing the project. Owing to the fragility of the Three Gorges Reservoir Area and the particularity of geographical location, its natural environment has always been the focus of attention. The unique advantage of these small towns in the reservoir area is likely to have a significant and positive impact on their natural environment and necessary public service facilities, which will lead to some differences between small towns scattered in the reservoir area and non-reservoir area. In such a unique geographical unit such as the reservoir area, whether tourist towns will be attached more importance so that their advantage in ecological livability will be more outstanding is also one of the main focuses of this paper. Also then, this paper divides Chongqing into the reservoir area and non-reservoir area and inspects whether the advantage in ecological livability of tourist towns is significant or not in different regions, that is, whether Hypothesis 4 of this paper is valid or not. Table 7 summarizes tourist towns located in the reservoir area and non-reservoir area.

**Table 7:** Chongqing reservoir area and non-reservoir area of three gorges project.

**Region**	**District and County**	**National tourist towns**	**Provincial tourist towns**
Reservoir area	Yu Bei, Ba Nan, Fu Lin, Jiang Jin, Chang Shou, Feng Jie, Yun Yang, Wu Shan, Wu Xi, Wan Zhou, Kai Zhou, Zhong Xian, Feng Du, Wu Long, Shi Zhu	Xing Long, Shi Bao, Lin Shi, Bai Di	Dong Wenquan, Xian Nvshan, Wu Lingshan, Chang Shouhu, Xin Sheng, Gan Ning
Non-reservoir area	Jiang Bei, Sha Pingba, Jiu Longpo, Nan An, Bei, Da Dukou, Qi Jiang, Wan Sheng, Da Zu, Qian Jiang, He Chuan, Yong Chuan, Nan Chuan, Rong Chang, Tong Nan, Liang Ping, Cheng Kou, Dian Jiang, Xiu Shan, You Yang, Peng Shui	Jing Guan, Wan-Ling, Bao Ding	You Shuihe, Hei Shan, Bai Shiyi, Cong Kan

Among the 15 districts and counties in the reservoir area, there are nine tourist towns, including four national tourist towns and five provincial ones. Among the 23 districts and counties in the non-reservoir area, there are eight tourist towns, including four national tourist towns and four provincial ones. Based on this division, this paper further empirically tests the vital issue of this paper, namely whether tourist towns are more ecologically livable than ordinary small towns. The detailed regression results are shown in Table 8.

**Table 8:** Results based on the division of reservoir and non-reservoir areas.

**Dependent variable: ecological livability of small towns**				
**Explanatory variables**	**(1)**	**(2)**	**(3)**	**(4)**
	**Reservoir area**		**Non-reservoir areas**	
Tourist towns	0.053*** (0.019)		0.026 (0.022)	
National tourist towns		0.082*** (0.024)		0.066** (0.032)
Provincial tourist towns		0.008 (0.030)		−0.012 (0.031)
Per capita GDP	0.066*** (0.005)	0.070*** (0.005)	0.078*** (0.003)	0.079*** (0.009)
Distance from district and county governments (logarithm)	−0.017*** (0.005)	−0.016*** (0.004)	−0.033*** (0.005)	−0.033*** (0.005)
Population urbanization (logarithm)	0.014*** (0.004)	0.014*** (0.005)	−0.001 (0.005)	−0.001 (0.005)
Land urbanization (logarithm)	−0.015*** (0.004)	−0.015*** (0.004)	0.007* (0.004)	0.007* (0.004)
Population size (logarithm)	−0.010 (0.007)	−0.009 (0.007)	−0.009 (0.006)	−0.008 (0.006)
Central town	0.007** (0.003)	0.007** (0.003)	0.014*** (0.004)	0.014*** (0.004)
Constant	0.210*** (0.021)	0.208*** (0.021)	0.261*** (0.018)	0.261*** (0.018)
Observation number	386	386	426	426
Log likelihood	540.392	542.266	573.053	574.613
LR test	36.56	40.30	103.32	106.44

Values in parentheses are standard deviation, and ***, **, and * indicate the significant levels of 1, 5, and 10%, respectively.

According to the results of column (1) in Table 8, nine tourist towns in the reservoir area are indeed more ecologically livable than ordinary small towns. Compared with the results in column (6) of Table 4, the coefficient is 0.53, which is significantly higher than that (0.39) in column (6) of Table 4, which indicates tourist towns located in the reservoir area are with a prominent advantage in ecological livability. The results of column (2) show that national tourist towns have a significant and massive advantage in ecological livability over ordinary small towns, whereas the provincial ones are no more ecologically livable. The results of columns (3) and (4) mean that eight tourist towns in the non-reservoir area are no more ecologically livable than ordinary small towns. Only the national tourist towns remain the advantage. From the discussed results, it is self-evident that Hypothesis 4 is valid. That is to say, compared with the non-reservoir area, tourist towns located in the reservoir area of the Three Gorges, which receive more financial resources and inclined policy support, still own the advantage in ecological livability.

## Conclusion

Cities have always been strongly linked with tourism and travel. The COVID-19 crisis may be an opportunity to rethink these relationships, help tourism recover, and in turn, shape more sustainable urban environments. In fact, for decades now, many cities have had to confront exactly the reverse effect of the COVID-19 standstill, struggling to deal with too much tourism, with residents' livability sometimes heavily impacted because of disruption, noise, or overcrowded transport. However, tourist towns are in a related industry, such as hotels or restaurants, and these are economically essential because fewer tourists and their less spending mean fewer jobs and strained city budgets.

Under the background that Rural Revitalization has become a national strategy of China, this paper takes tourist towns as the research object and analyzes the core issue that whether the financial support and tourism aura endow tourist towns with the advantage in ecological livability over ordinary small towns. Based on the *Basic Data of Towns and Townships in Chongqing 2016* and other relevant data, this paper conducts empirical tests on the core issue. The main conclusions are as follows: Firstly, according to the results of benchmark regression, compared with ordinary small towns, tourist towns are more ecologically livable. This result proves that the policy of tourist towns indeed endows those towns with the advantage of ecological livability. Secondly, both national and provincial tourist towns are more ecologically livable than ordinary small towns, but the advantage of national tourist towns is more prominent. This paper divides tourist towns into two categories: national tourist towns and provincial ones, and then, the study on the core issue is extended. The results indicate that the coefficients and significance levels of both national and provincial tourist towns are stable. Thirdly, in regions with low tourism dependence, tourist towns are not more ecologically livable than ordinary small towns, whereas in regions with high tourism dependence, the advantage of tourist towns is still very significant. Lastly, tourist towns in the reservoir area of the Three Gorges Project are more ecologically livable than ordinary small towns.

In contrast, the advantage of the 23 tourist towns in the non-reservoir area is not significant. Also, national tourist towns in the reservoir area and non-reservoir area are more ecologically livable than ordinary small towns. In contrast, the provincial-level towns do not have such an advantage. The COVID-19 pandemic shows us to integrate nature-based solutions into urban planning as a means to preserve local ecosystems while enhancing local resilience and improving residents' quality of life. The COVID-19 crisis in 2020 demonstrates that current systems and communities are not resilient enough. It is essential to use nature-based solutions that can improve social, economic, and environmental benefits for inclusive urban development and ensures that its residents have access to good quality, safe, and attractive open spaces. At this stage, the green infrastructures should be developed in the leading public spaces where residents and tourists enjoy their leisure time. Building multipurpose cities, such as Chongqing, can provide more economically resilient cities and regions beyond COVID-19.

Different from developed countries, the urbanization rate of developing ones is quite low. Also, there are still millions of people living in towns. During the process of urbanization, how to improve the efficiency of resource allocation is a core issue. In this sense, this paper's conclusion has essential policy significance, and it is only limited to China. Therefore, future studies can focus on the case of other large developing economies, such as Brazil and India.

## Data Availability Statement

The raw/processed data required to reproduce these findings cannot be shared at this time as the data are under the supervision of Chongqing Housing and Urban Rural Development Commission. The list of towns can be found at: Chongqing Municipal Government (2). People's Government Portal. Ministry of Housing and Urban-Rural Construction of the People's Republic of China (1). Central Government Portal.

## Author Contributions

KS: data curation, investigation, supervision, and writing—original draft preparation. CZ: conceptualization, methodology, software, and visualization.

## Conflict of Interest

The authors declare that the research was conducted in the absence of any commercial or financial relationships that could be construed as a potential conflict of interest.

## Footnotes

^1^China's 31 administrative regions are composed of 27 provinces and 4 municipalities directly under the Central Government (Beijing, Shanghai, Tianjin, and Chongqing). The municipality directly under the Central Government is a provincial administrative region and plays an important role in politics, economy, and culture.
